# The History and Introduction of Anesthesia in Surgery*Abstract of a lecture delivered at the Medical Department, University of Buffalo, October, 16, 1896. Published in full in *Buffalo Medical Journal*, November.

**Published:** 1896-12

**Authors:** Roswell Park

**Affiliations:** Buffalo, N. Y., Professor of Surgery


ATLANTA

Medical and Surgical Journal.

VOL. XIII. DECEMBER, 1896. No. 10.

LUTHER B. GRANDY, M.D., MANAGING EDITOR.

M. B. HUTCHINS, M.D., BUSINESS MANAGER.

COLLABORATORS:
A. W. CALHOUN, M.D., LL.D., VIRGIL O. HARDON, M.D., FLOYD W. McRAE, M.D., DUNBAR ROY, M.D., AND HENRY R. SLACK, PH.M., M.D., LAGRANGE, GA.

### ORIGINAL COMMUNICATIONS.

### THE HISTORY AND INTRODUCTION OF ANESTHESIA IN SURGERY.*

### IN COMMEMORATION OF THE SEMI-CENTENNIAL OF THE INTRODUCTION OF ETHER AS AN ANESTHETIC AGENT.

BY ROSWELL PARK, A.M., M.D., BUFFALO, N. Y.,
Professor of Surgery.

Fifty years ago to-day—that is to say, on the 16th of October, 1846—there occurred an event which marks as distinct a step in human progress as almost any that could be named by the erudite historian. I refer to the demonstration of the possibility of alleviating pain during surgical operations. Had this been the date of a terrible battle, on land or sea, with mutual destruction of thousands of human beings, the date itself would have been signalized in literature, and would have been impressed upon the memory of every schoolboy, while the names of the great military murderers

* Abstract of a lecture delivered at the Medical Department, University of Buffalo, October, 16, 1896. Published in full in *Buffalo Medical Journal*, November.

who commanded the opposing armies would have been emblazoned upon monuments and the pages of history. But this event was merely the conquest of pain and the alleviation of human suffering, and no one who has ever served his race by contributing to either of these results has been remembered beyond his own generation or outside the circle of his immediate influence. Such is the irony of fate. The world erects imposing monuments or builds tombs, like that of Napoleon, to the memory of those who have been the greatest destroyers of their race; and so Cæsar, Hannibal, Genghis Khan, Richard the Lionhearted, Gustavus Vasa, Napoleon, and hundreds of other great military murderers have received vastly more attention, because of their race-destroying propensities and abilities, than if they had ever fulfilled fate in any other capacity. But the men like Sir Spencer Wells, who has added his 40,000 years of life to the total of human longevity; or like Sir Joseph Lister, who has shown our profession how to conquer that arch enemy of time past, surgical sepsis; or like Morton, who first publicly demonstrated how to bring on a safe and temporary condition of insensibility to pain, are men more worthy, in our eyes, of lasting fame, and much greater heroes of their times, and of all time, yet are practically unknown to the world at large, to whom they have ministered in such an unmistakable and superior way.

From all time it has been known that many different plants and herbs contained principles which were narcotic, stupefying or intoxicating. These properties have especially been ascribed to the juices of the poppy, the deadly nightshade, henbane, the Indian hemp and the mandragora, which for us now is the true mandrake, whose juice has long been known as possessing soporific influence. Ulysses and his companions succumbed to the influence of *nepenthe;* and, nineteen hundred years ago, when crucifixion was a common punishment of malefactors, it was customary to assuage their last hours upon the cross by a draught of vinegar with gall or myrrh, which had real or supposititious narcotic properties. Even the prophet Amos, seven hundred years before the time of Christ, spoke of such a mixture as this as "the wine of the condemned," for he says, in rehearsing the iniquities of Israel by which they had incurred the anger of the Almighty: "And they lay themselves down upon the clothes laid to pledge by every altar, and they drink the wine of the condemned in the house of their God" (Chap. II., verse 8), meaning thereby undoubtedly that these people, in their completely demoralized condition, drank the soporific draught kept for criminals. Herodotus mentions a habit of the Scythians, who employed a vapor generated from the seed of the hemp for the purpose of producing an intoxication by inhalation. Narcotic lotions were also used for bathing the people about to be operated upon. Pliny, who perished at the destruction of Herculaneum, A. D. 79, testified to the soporific power of the preparations made from mandragora upon the faculties of those who drank it. He says: "It is drunk against serpents and before cuttings and puncturings, lest they should be felt." He also describes the indifference to pain produced by drinking a vinous infusion of the seeds of eruca, called by us the rocket, upon criminals about to undergo punishment. Dioscorides relates of mandragora that "some boil down the roots in wine to a third part, and preserve the juice thus procured, and give one cyathus of this to cause the insensibility of those who are about to be cut or cauterized." One of his later commentators also states that wine in which mandragora roots have been steeped "does bring on sleep and appease pain, so that it is given to those who are to be cut, sawed or burnt in any parts of their body, that they may not perceive pain. Apulieus, about a century later than Pliny, advised the use of the same preparation. The Chinese, in the earlier part of the century, gave patients preparations of hemp, by which they became completely insensible, and were operated upon in many ways. This hemp is the cannabis indica which furnishes the *hasheesh* of the Orient and the intoxicating and deliriating *bhang*, about which travelers in the East used to write so much. In Barbara, for instance, it was always taken, if possible, by criminals condemned to suffer mutilation or death.

According to the testimony of medieval writers, knowledge of these narcotic drugs was practically applied during the last of the Crusades, the probability being that the agent principally employed was this same hasheesh. Hugo di Lucca gave a complete formula for the preparation of the mixture, with which a sponge was to be saturated, dried, and then, when wanted, was to be soaked in warm water, and afterward applied to the nostrils, until he who was to be operated upon had fallen asleep; after which he was aroused with the vapor of vinegar.

Strangely enough, the numerous means of attaining insensibility, then more or less known to the common people, and especially to criminals and executioners, do not appear to have found favor for use during operations. Whether this was due to unpleasant after-effects, or from what reason, we are not informed. Only one or two surgical writers beside Guy de Chauliac (1498) refer in their works to agents for relief of pain, and then almost always to their unpleasant effects, the danger of producing asphyxiation, and the like. Ambrose Paré wrote that preparations of mandragora were formerly used to avert pain. In 1579, an English surgeon, Bulleyn, affirmed that it was possible to put the patient into an anesthetic state during the operation of lithotomy, but spoke of it as a "terrible dream." One Meisner spoke of a secret remedy used by Weiss, about the end of the XVII. century, upon Augustus II., king of Poland, who produced therewith such perfect insensibility to pain that an amputation of the royal foot was made without suffering, even without royal consent. The advice which the Friar gave Juliet regarding the distilled liquor which she was to drink, and which should presently throw her into a cold and drowsy humor, although a poetic generality, is Shakespeare's recognition of a popular belief.

Of course, of all the narcotics in use by educated men, opium has been, since its discovery and introduction, the most popular and generally used. Surgeons of the last century were accustomed to administer large doses of it shortly before an operation, which, if serious, was rarely performed until the opiate effect was manifested. Still, in view of its many unpleasant after-effects, its use was restricted, so far as possible, to extreme cases.

Baron Larrey, noticing the benumbing effect of cold upon wounded soldiers, suggested its introduction for anesthetic purposes, and Arnott, of London, systematised the practice, by recommending a freezing mixture of ice and salt to be laid directly upon the part to be cut. Other surgeons were accustomed to put their patients into a condition of either alcoholic intoxication or alcoholic stupor. Long-continued pressure of a part was also practiced by some, by which a limb could, as we say, be made to go to sleep. A few others recommended to produce faintness by excessive bleeding. It was in 1776 that the arch-fraud Mesmer entered Paris and began to initiate people into the mysteries of what he called *animal magnetism*, which was soon named mesmerism, after him. Thoroughly degenerate and disreputable as he was, he nevertheless taught people some new truths, which many of them learned to their sorrow, while in the hospitals of France and England severe operations were performed upon patients thrown into a mesmeric trance, and without suffering on their part.

In 1799, Sir Humphry Davy, being at that time an assistant in the private hospital of Dr. Beddoes, which was established for treatment of disease by inhalation of gases, and which he called The Pneumatic Institute, began experimenting with nitrous oxide gas, and noticed its exhilarating and intoxicating effects; also the relief from pain which it afforded in headache and toothache. As the results of his reports, a knowledge of its properties was diffused all over the world, and it was utilized both for amusement and exhibition purposes. Davy even wrote as follows of this gas: "As nitrous oxide, in its extensive operation, appears capable of destroying physical pain, it may probably be used with advantage during surgical operations in which no great effusion of blood takes place."

It is not at all unlikely that Colton and Wells, to be soon referred to, derived encouragement, if not incentive, from these statements of Davy. Nevertheless, Velpeau, perhaps the greatest French surgeon of his day, wrote in 1839, that "to escape pain in surgical operations is a chimera which we are not permitted to look for in our day."

Sulphuric ether, as a chemical compound, was known from the XIII. century, for reference was made to it by Raymond Lully. It was first spoken of by the name of ether by Godfrey in the Transactions of the London Royal Society in 1730, while Isaac Newton spoke of it as the ethereal spirits of wine. During all of the previous century it was known as a drug, and allusion to its inhalation was made in 1795 in a pamphlet, probably by Pearson.

Beddoes, in 1796, stated that "it gives almost immediate relief, both to the oppression and pain in the chest, in cases of pectoral catarrh." In 1815, Nysten spoke of inhalation of ether as being common treatment for mitigating pain in colic, and in 1816 he described an inhaler for its use. As early as 1812 it was often inhaled for experiment or amusement, and so-called "ether frolics" were common in various parts of the country. This was true, particularly for our purpose, of the students of Cambridge and of the common people in Georgia in the vicinity of Long's home. It probably is for this reason that a host of claimants for the honor of the discovery appeared so soon as the true anesthetic properties of the drug were demonstrated.

There probably is every reason to think that, either by accident or design, a condition of more or less insensibility to pain had been produced between 1820 and 1846, by a number of different people, educated and ignorant, but that no one had the originality or the hardihood to push these investigations to the point of determining the real usefulness of ether. This was partly from ignorance, partly from fear, and partly because of the generally accepted impossibility of producing safe insensibility to pain. So, while independent claim sprang up from various sources, made by aspirants for honors in this direction, it is undoubtedly as properly due to Morton to credit him with the introduction of this agent as an anesthetic as to credit Columbus with the discovery of the New World, in spite of certain evidences that some portions of the American continent had been touched upon by adventurous voyagers before Columbus ever saw it.

The terms "anesthesia" and the adjective "anesthetic" were suggestions of Dr. Oliver Wendell Holmes, who early proposed their use to Dr. Morton in a letter which is still preserved. He suggests them with becoming modesty, advises Dr. Morton to consult others before adopting them, but, nevertheless, states that he thinks them apt for that purpose. The word anesthesia, therefore, is just about of the same age as the condition itself, and it, too, deserves commemoration upon this occasion.

As one reads the history of anesthesia, which has been written up by a number of different authors, each, for the most part, having some particular object in view, or some particular friend whose claims he wishes especially to advocate, he may find mentioned at least a dozen different names of men who are supposed to have had more or less to do with this eventful discovery. But, for all practical purposes, one may reduce the list of claimants for the honor to four men, each of whose claims I propose to briefly discuss. These men were Long, Wells, Jackson and Morton. Of these four, two were dentists and two practicing physicians, to whom fate seems to have been unkind, as it often is, since three of them at least died a violent or distressing death, while the fourth lived to a ripe old age, harassed at almost every turn by those who sought to decry his reputation or injure his fortunes.

Crawford W. Long was born in Danielsville, Ga., in 1816. In 1839 he graduated from the Medical Department of the University of Pennsylvania. In the part of the country where Long settled it was a quite common occurrence to have what were known as "ether frolics" at social gatherings, ether being administered to various persons to the point of exhilaration, which in some instances was practically uncontrollable. Long's friends claim that he had often noticed that when the ether effect was pushed to this extent the subjects of the frolic became oblivious to minor injuries, and that these facts, often noticed, suggested to his mind the use of ether in surgical operations. There is good evidence to show that Long first administered ether for this purpose on the 30th of March, 1842, and that on June 6th he repeated this performance on the same patient; that in July he amputated a toe for a negro boy, but that the fourth operation was not performed until September of 1843. In 1844 a young man, named Wilhite, who had helped to put a colored boy to sleep at an ether frolic in 1839, became a student of Dr. Long's, to whom Long related his previous experiences. Long had never heard of Wilhite's episode, but had only one opportunity, in 1845, to try it, again upon a negro boy. Long lived at such a distance from railroad communication (130 miles) as to have few advantages, either of practice, observation, or access to literature. Long made no public mention of his use of ether until 1849, when he published "An Account of the First Use of Sulphuric Ether by Inhalation as an Anesthetic in Surgical

Operations," stating that he first read of Morton's experiments in an editorial in the *Medical Examiner* of December, 1846, and again later; on reading which articles he determined to wait before publishing any account of his own discovery, to see whether any one else would present a prior claim. No special attention was paid to Long's article, as it seemed that he merely desired to place himself on record. There is little, probably no reasonable, doubt as to Long's priority in the use of ether as an anesthetic, although it is very doubtful if he carried it, at least at first, to its full extent. Nevertheless Long was an isolated observer, working entirely by himself, having certainly no opportunity and apparently little ambition to announce his discovery, and having no share in the events by which the value of ether was made known to the world. Long's strongest advocate was the late Dr. Marion Sims, who made a strong plea for his friend, and yet was not able to successfully establish anything more than has just been stated.

Horace Wells was born in Hartford, Vt., in 1815. In 1834 he began to study dentistry in Boston, and after completing his studies began to practice in Hartford, Ct. He was a man of no small ingenuity, and devised many novelties for his work. In December, 1844, he listened to a lecture delivered by Dr. Colton, who took for his subject nitrous oxide gas, the amusing effects of which he demonstrated to his audience upon a number of persons who visited the platform for that purpose. Wells was one of these. Wells, moreover, noticed that another young man, who bruised himself while under its influence, said afterward that he had not hurt himself at all. Wells then stated to a bystander that he thought that if one took enough of that kind of gas he could have a tooth extracted and not feel it. He at once called upon a neighboring dentist friend and made arrangements to test the anesthetic effects of the gas upon himself the next morning. Accordingly Colton gave him the gas, and Riggs, the friend, extracted the tooth; and Wells, returning to consciousness, assured them both that he had not suffered a particle of pain. He began at once to construct an apparatus for its manufacture. Dr. Marcey, of Hartford, then informed Wells that while a student at Amherst he and others had often inhaled nitrous oxide as well as the vapor of ether, for amusement, and suggested to Wells to try ether. After a few trials, however, it was found more difficult to administer, and Wells accordingly resolved to adhere to gas alone. This was in 1844, two years after Long's obscure experiments, of which, of course, they were ignorant. In 1845, Wells visited Boston for the purpose of introducing his discovery, and among others called upon his former partner, Morton, trying to establish the use of the gas. He soon became discouraged, however, and returned to Hartford, resuming his practice. There he continued to use gas for about two years, but failed to secure its introduction into general surgery, owing to prejudice and ignorance on the part of dentists and physicians alike.

Wells's claims have been advocated by many of his fellow-citizens, and in Bushnell Park, in Hartford, stands a monument erected by the city and State, dedicated to Horace Wells, "who discovered anesthesia, November, 1844."

C. T. Jackson was born in Plymouth, Mass., in 1805. He graduated in the Harvard Medical School in 1829, after which he went abroad, where he remained for several years, made the acquaintance of the most distinguished men, experimented in general science, electricity and magnetism, and even devised a telegraphic apparatus, similar to that which Morse patented a year later. Returning, in 1835, he opened in Boston a laboratory for instruction in analytical chemistry, the first of its kind in the country. In 1846 and 1847 he was much aroused by Morton's experiments with sulphuric ether, and claimed even that he had suggested the use of ether to Morton, claiming also that he had himself been relieved of an acute distress by inhalation of ether vapor, and that it was from reflection on the phenomena presented in his own case that the possibility of its use for relief of pain during surgical operations suggested itself to him. This led to a triangular conflict for the priority of discovery between Wells, Jackson and Morton, each claiming the honor for himself. Wells's health soon gave way. He went abroad and got recognition from the French Institute and the Paris Academy of Sciences, which did not, however, indorse his claim as discoverer nor accept nitrous oxide as an anesthetic. Wells returned to find that Morton was on the tide of popular favor, the public having indorsed ether as the only reliable anesthetic. His mind became unbalanced, and in a fit of temporary aberration he ended his own life in a prison cell in New York city in 1848.

In 1873, Jackson's mind gave way, and after seven years of confinement in an asylum he died in 1880, at the age of 75, having been the recipient of many honors from foreign potentates and learned societies.

William T. G. Morton was born in Charleston, Mass., in 1819. After a disastrous experience in business he was sent to Baltimore in 1840 and began the study of dentistry. In 1841 he entered the dental office of Horace Wells as student and assistant, becoming a partner in 1842. In 1843 the partnership was dissolved, Wells removing to Hartford, as before stated. Morton, ambitious for a medical degree, entered his name as a student in the office of Charles T. Jackson, in 1844, and the same year matriculated in the Harvard Medical School, though he never graduated. Having learned through Wells, of the latter's successful use of nitrous oxide gas, but not knowing how to make it, he sought the advice of Dr. Jackson, who informed him that its preparation entailed considerable difficulty, and inquired for what purpose he wanted it. On Morton's replying that he wished to use it to make patients insensible to pain, Jackson suggested the use of sulphuric ether, as Marcey had suggested it to Wells, two years previously, saying that it would produce the same effect and did not require any apparatus. Jackson also told Morton of the ether frolics common at Cambridge among the students. That same evening, September 30, 1846, Morton administered ether to a patient and extracted a tooth for him without pain. The next day he visited the office of a patent lawyer, for the purpose of securing a patent upon the new discovery. This lawyer ascertained that Jackson had been intimately connected with its suggestion, and came to the conclusion that a patent could not safely issue to either one independently of the other. But Jackson being a member of the State Medical Society, against whose ethical code it is to patent discoveries that pertain to the welfare of patients, and fearing the censure of his colleagues, agreed at once to assign his right over to Morton, receiving in return a 10 per cent. commission upon all that the latter made out of it. Morton, as a dentist, having no more compunction then than dentists have now upon the securement of a patent,—in other words, being actuated by no fine ethical scruples, —secured the patent, and then called upon Dr. J. Mason Warren, one of the surgeons in the Massachusetts General Hospital. Warren promised his coöperation and appointed the 16th of October, 1846, for the first public trial. Upon this occasion the clinic room was filled with visitors and students, when Morton placed the young man under the influence of his "letheon," as he called it then; after which Warren removed a tumor from his neck. The trial was most successful. Another took place on the following day, and on November 7th an amputation and an excision of the jaw were made, both patients being under the influence of letheon and oblivious to pain. At this time the nature of the anesthetic agent was kept a secret, the vapor of ether being disguised by aromatics so as not to be recognized by any one present.

True to the highest traditions of their craft the staff of the Massachusetts General Hospital now met and declined to make further use of a drug whose composition was thus kept secret. It was then that Morton revealed the exact nature of it as sulphuric ether, disguised with aromatic oils.

In 1846 an English patent was obtained.

Morton soon began the attempt to sell office rights, as do the dentists of to-day, while the medical profession was then, as ever, antagonistic to patents, holding them to be subversive of general good. His patent was soon opposed and then generally infringed upon. Litigation followed without end and the government stultified itself by refusing to recognize the validity of the patent issued by itself. And so, without any compensation to the discoverer, ether soon came into general use in this country as abroad. While receiving many congratulations from friends and humanitarians, Morton's success aroused the jealousy of some of his professional brethren, among them one Dr. Flagg, who commenced a terrible onslaught upon the new application of ether and its promoter. By his machinations a meeting of Boston dentists was called and a committee of twelve appointed to make a formal protest against anesthesia. This committee published a manifesto in the *Boston Daily Advertiser*, in which all sorts of untoward effects and unpleasant results were attributed to the new anesthetic. This proclamation was spread broadcast, and did Morton, for the time, very much harm.

These and similar statements created a very strong prejudice against Morton, who, in December, 1846, sent to Washington, to a nephew of Dr. Warren, to endeavor to urge upon the government the advantages of employing ether in the army during the Mexican war, then in progress. The chief of the Bureau of Medicine and Surgery reported that the article might be of some service for use in large hospitals, but did not think it expedient for the department to incur any expense by introducing it into the general service; while the acting surgeon-general believed that the highly volatile character of the substance itself made it ill-adapted to the rough usage it would necessarily encounter upon the field of battle, and accordingly declined to recommend its use.

In January of 1853, Morton demonstrated at the infirmary in Washington, before a congressional committee and others, the anesthetic effect of ether, which he continued through a dangerous and protracted surgical operation. This was the result of a challenge to compare the effects of nitrous oxide and those of ether, the advocates of the former not putting in an appearance.

The balance of Morton's life seems to have been spent in continued jangles. The government, having repudiated its own patent, was repeatedly besought by memorials and through the influence of members of Congress to bestow some testimonial upon or make some money return to Morton for his discovery. Several times he came near a realization of his hopes in this respect, when the action of some of his enemies or the termination of a congressional session, or some other accident, would doom him again to disappointment. The pages of evidence that were printed, the various reports issued through or by government officers, the memorials addressed from various individuals and societies, if all printed together would make a large volume; but all of these were of no avail. Morton spent all his means, as he spent his energies and time, in futile endeavor to get pecuniary recognition of his discovery, but was doomed to disappointment. He seemed alike a victim of unfortunate circumstances and of treachery and animosity upon the part of his opponents. Especially did the fight wage warm between him and his friends and Jackson. Plots to ruin his business were repeatedly hatched and his life was made miserable in many ways. Mere temporary sops to wounded vanity and impaired fortune were the honorary degrees and the testimonials that came to him from various institutions of learning and foreign societies. In 1850 both Morton and Jackson received from the French Academy prizes valued at 2,500 francs each. Finally, Morton fell into a state of nervous prostration, suffered from anxiety and insomnia, and in a fit of temporary aberration exposed himself in Central Park, New York, became unconscious and was taken to St. Luke's hospital, dying just as he reached the institution, on the 15th day of July, 1868. In Mount Auburn cemetery, in Boston, there stands a beautiful monument to William T. G. Morton, bearing this inscription : "Inventor and revealer of anesthetic inhalation, before whom in all time surgery was agony ; by whom pain in surgery was averted and annulled; since whom science has control of pain."

Summing up, then, the claims of our four contestants in the light of a collected history of the merits of each, it would appear that Wells first made public use of nitrous oxide gas for limited purposes, but failed to introduce it into general professional use. That Long, in an isolated rural practice, a few times used ether, with which he produced probably only partial insensibility to pain, and that he had apparently discontinued its use before learning of Morton's researches. That Jackson made no claim to the use of the agent on his own part, but simply having suggested it to Morton. And finally that Morton quickly accepted the suggestion, made careful and scientific use thereof, but especially, and above all other things, first *demonstrated* to the world at large the capability and the safety of this agent as an absolute, reliable and efficient anesthetic. So, though Morton permitted his cupidity to run away with finer ethical considerations, and attached a higher pecuniary than humanitarian value to sulphuric ether, he, nevertheless, must be generally credited with having, to use the modern expression, "promoted" its introduction, and to have shown to the world at large what an inestimably valuable therapeutic agent had been added to our resources for the control of pain.

